# Triple Genetic Alterations (EGFR Mutation, ALK Fusion, and MET Polysomy) in Gynecological Metastasis of Lung Adenocarcinoma Patient with Acquired Resistance to Furmonertinib: A Case Report

**DOI:** 10.3390/curroncol33100613

**Published:** 2026-10-12

**Authors:** Qinglong Jiang, Rui Sun, Haiyan Xu, Yanfeng Wang

**Affiliations:** Department of Comprehensive Oncology, National Cancer Center/National Clinical Research Center for Cancer/Cancer Hospital, Chinese Academy of Medical Sciences and Peking Union Medical College, Beijing 100021, China; jqinglongsdu@126.com (Q.J.); moonlet@126.com (R.S.)

**Keywords:** epidermal growth factor receptor, next-generation sequencing, fluorescence in situ hybridization, furmonertinib, ensartinib, uterine metastasis, ALK fusion

## Abstract

For advanced non-small cell lung cancer (NSCLC) with epidermal growth factor receptor (EGFR) mutations, targeted therapy is the standard first-line treatment, but acquired resistance and disease progression are inevitable. Identification of resistance mechanisms is crucial for guiding subsequent anti-tumor therapy. We report a 57-year-old female patient with advanced lung adenocarcinoma harboring an EGFR exon 21 L858R mutation. The patient experienced disease progression with rare gynecological metastasis after 16 months of first-line furmonertinib therapy. Next-generation sequencing (NGS) of the uterine metastatic lesion identified concurrence of EGFR mutation and ALK fusion, while fluorescence in situ hybridization (FISH) confirmed MET polysomy. The patient exhibited intolerance to the combination therapy of glumetinib plus furmonertinib. Subsequently, the patient received a combination of furmonertinib and the ALK-TKI ensartinib, which was assessed as a partial response (PR) at the first restaging evaluation (1 month after treatment initiation), with favorable safety and tolerability. Secondary ALK fusion may represent a rare but clinically meaningful resistance mechanism in EGFR-mutant NSCLC. Furthermore, while gynecological metastasis from lung cancer is uncommon, clinicians should be alert to this possibility in patients presenting with relevant symptoms, with pelvic examination conducted as needed.

## Simple Summary

This case report tells the story of a 57-year-old woman with rare uterine metastasis of EGFR L858R-mutant advanced lung adenocarcinoma. The patient progressed after 16 months of first-line furmonertinib. NGS of uterine lesions detected concurrent EGFR and ALK alterations, and FISH verified MET polysomy. This is a rare case report of uterine metastasis after furmonertinib resistance with three simultaneous gene aberrations. Repeated genetic testing is vital for progressive patients to guide subsequent combination targeted treatments.

## 1. Introduction

Approximately 85% of lung cancers are non-small cell lung cancer (NSCLC), and most patients are diagnosed with distant metastasis [1]. Common metastatic sites include the brain, bone, liver, and adrenal glands. Metastasis to the female reproductive system, particularly uterine metastasis, is relatively rare [2].

The treatment of NSCLC has entered the era of precision medicine. EGFR-mutant NSCLC accounts for up to 50% of cases in the East Asian population [3]. Classical EGFR alterations, including exon 19 deletions and exon 21 L858R point mutations, are highly sensitive to EGFR tyrosine kinase inhibitors (TKIs). Although third-generation EGFR-targeted therapy has demonstrated superior efficacy as first-line treatment for advanced NSCLC, acquired resistance remains inevitable. Resistance mechanisms are highly heterogeneous, including on-target alterations (e.g., EGFR C797S, L718Q) as well as off-target mechanisms such as bypass pathway activation (e.g., MET amplification/overexpression, HER2 activation) and histologic transformation to small-cell lung cancer [4].

MET gene amplification constitutes a key resistance mechanism in EGFR-mutant lung cancer and manifests in two distinct forms: focal amplification and polysomy. Both alterations upregulate MET mRNA levels, resulting in elevated MET protein expression and augmented MET pathway signaling upon activation. In lung cancer, focal MET amplification serves as a more robust oncogenic driver compared with polysomy. According to published data, the incidence of MET amplification is 7–15% upon acquired resistance to first-line osimertinib, whereas the corresponding rate ranges from 5% to 50% after second-line osimertinib failure [5]. Anaplastic lymphoma kinase (ALK) fusion is another key therapeutic target in NSCLC, occurring in approximately 5% of patients [6]. EGFR and ALK alterations are generally considered mutually exclusive; synchronous or sequential co-occurrence of both aberrations in a single patient is rare, and triple concurrent genomic alterations are exceptionally uncommon. Previous studies have reported that approximately 1–3% of patients with acquired resistance to EGFR TKIs develop secondary ALK fusion [7]. The optimal management strategy for patients with multigene co-mutations remains unclear, especially with regard to combination targeted therapy. Here, we report a case of advanced EGFR-mutant lung adenocarcinoma with rare gynecological metastasis that progressed under furmonertinib treatment.

## 2. Case Presentation

A 57-year-old female patient presented in April 2024 with chest distress and dyspnea. Chest computed tomography (CT) demonstrated a 3.2 cm × 3.1 cm mass adjacent to the mediastinum in the right upper lobe lung, accompanied by multiple mediastinal lymph node metastases. Brain magnetic resonance imaging (MRI) revealed multiple brain metastases, and bone scan showed multiple spinal metastases. Bronchoscopic biopsy confirmed lung adenocarcinoma. Molecular profiling of tissue and plasma identified an EGFR exon 21 L858R mutation, with no alterations detected in ALK, ROS1, RET, MET, KRAS, and NTRK. In May 2024, the patient was treated with furmonertinib 80 mg once daily combined with denosumab 120 mg monthly. One month later, the pulmonary lesion exhibited a marked reduction in size on follow-up computed tomography, with the residual lesion measuring 2.2 × 2.0 cm. Follow-up brain MRI showed that the brain metastatic lesion was reduced in size from 0.7 cm × 0.7 cm to 0.6 cm × 0.3 cm. The therapeutic response was evaluated as partial response (PR) according to the RECIST criteria. No significant adverse events were observed, and regular follow-up evaluations confirmed sustained PR.

**Figure 1:**
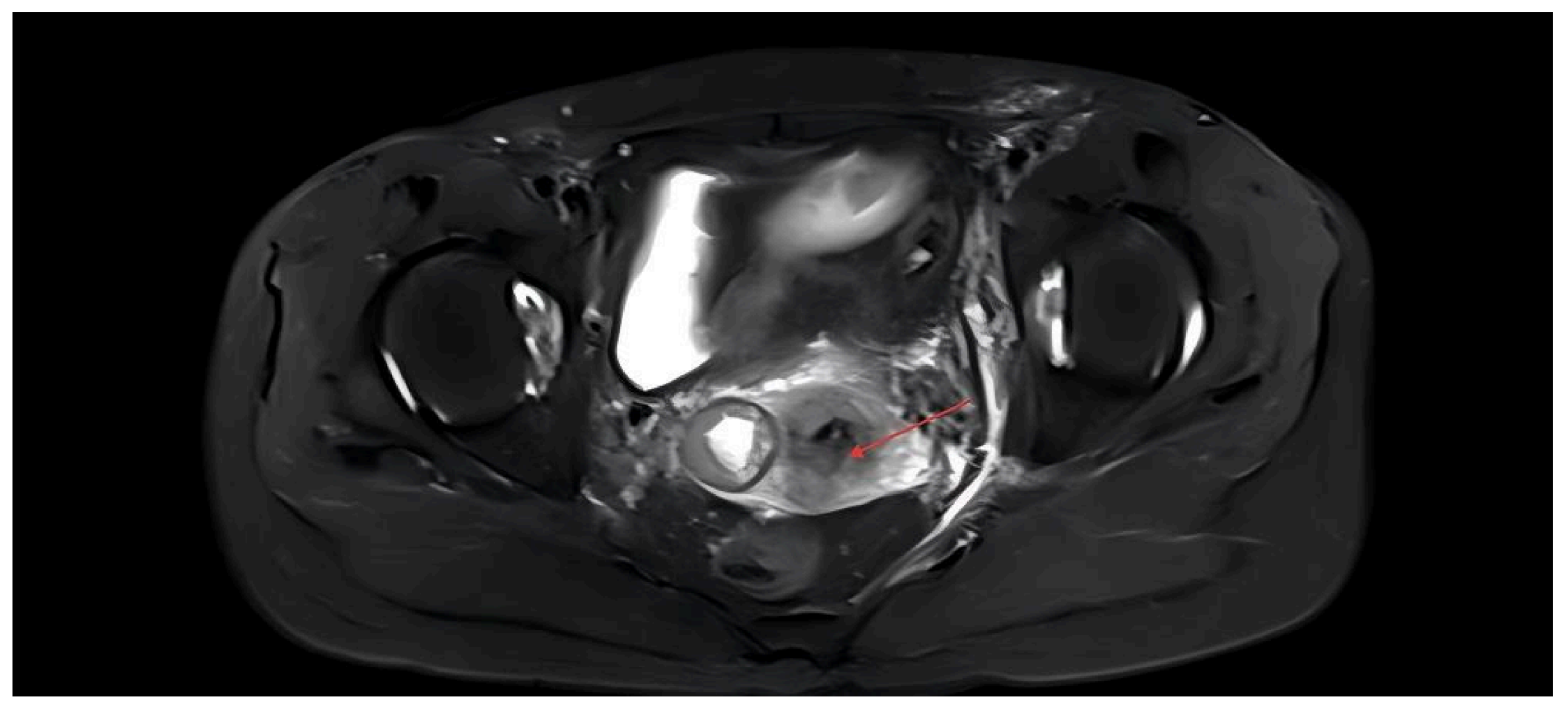
Pelvic magnetic resonance imaging (MRI) performed on 12 October 2025 showed metastasis in the uterus and pelvic lymph nodes. Diffuse myometrial thickening with abnormal signal and DWI restriction. The dominant lesion is about 2.7 × 2.5 cm (red arrow), invading vaginal fornix.

**Figure 2:**
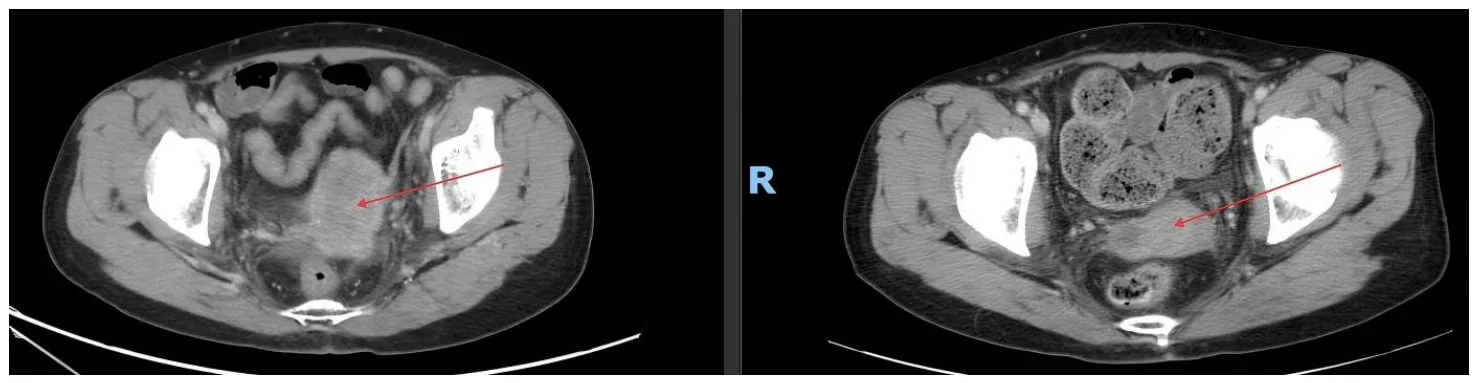
After targeted therapy, follow-up contrast-enhanced CT shows a significant reduction in pelvic metastatic lesions. (**Left**) panel: Pre-treatment metastatic uterine lesion in pelvic cavity, measuring approximately 6.5 × 4.8 cm (red arrow, acquired on 27 October 2025). (**Right**) panel: One month after combined targeted therapy, the metastatic uterine lesion decreased to 2.4 × 2.1 cm in size (red arrow, acquired on 3 December 2025).

**Figure 3:**
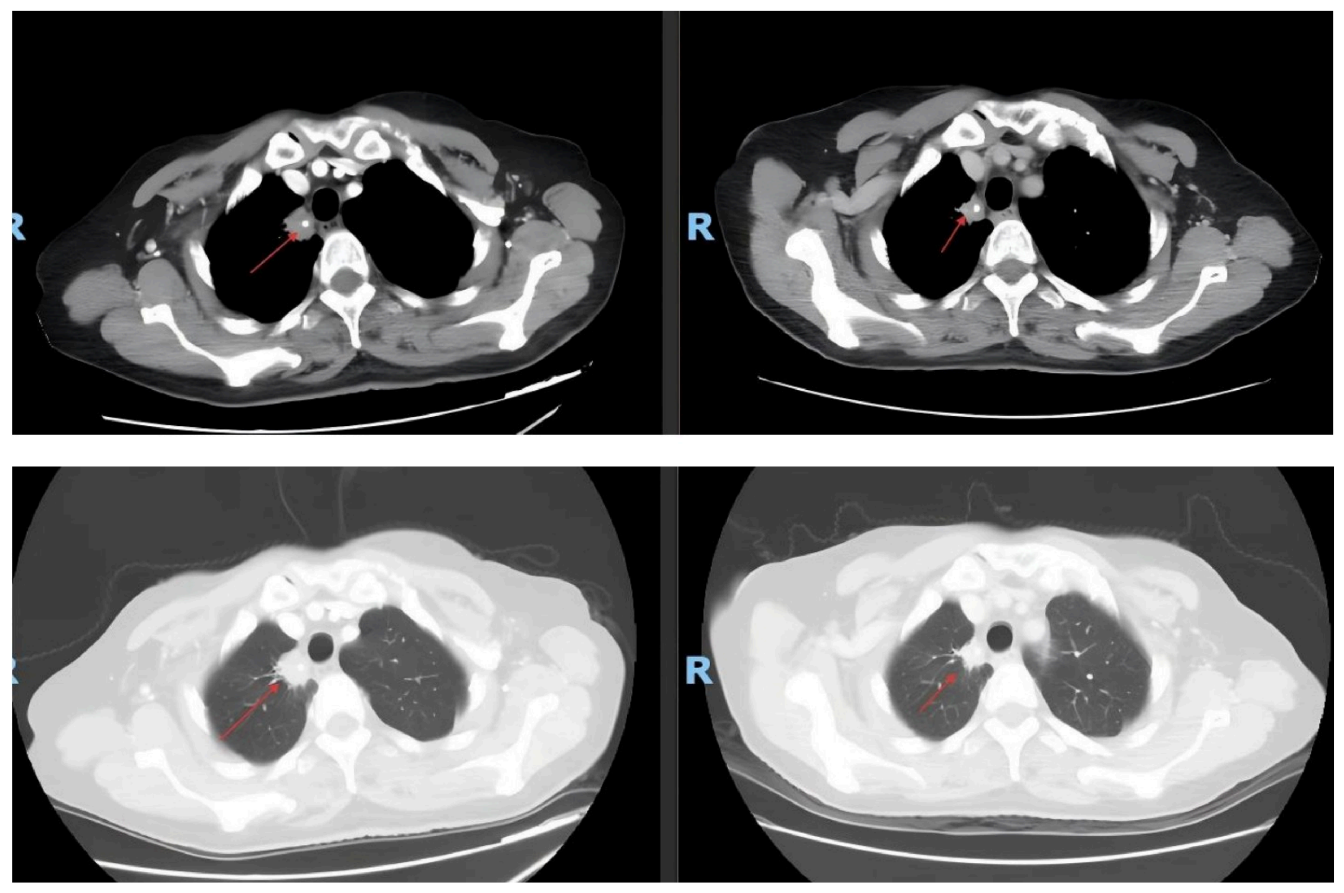
After dual-targeted therapy with furmonertinib combined with ensartinib, the primary pulmonary lesions in the patient were further reduced compared with the previous examination. (**Left**) The pulmonary lesion prior to dual-targeted therapy (red arrow, acquired on 27 October 2025); (**Right**) The pulmonary lesion after one month of dual-targeted therapy (red arrow, acquired on 3 December 2025); (**upper**) CT mediastinal window; (**Lower**) CT lung window.

**Figure 4:**
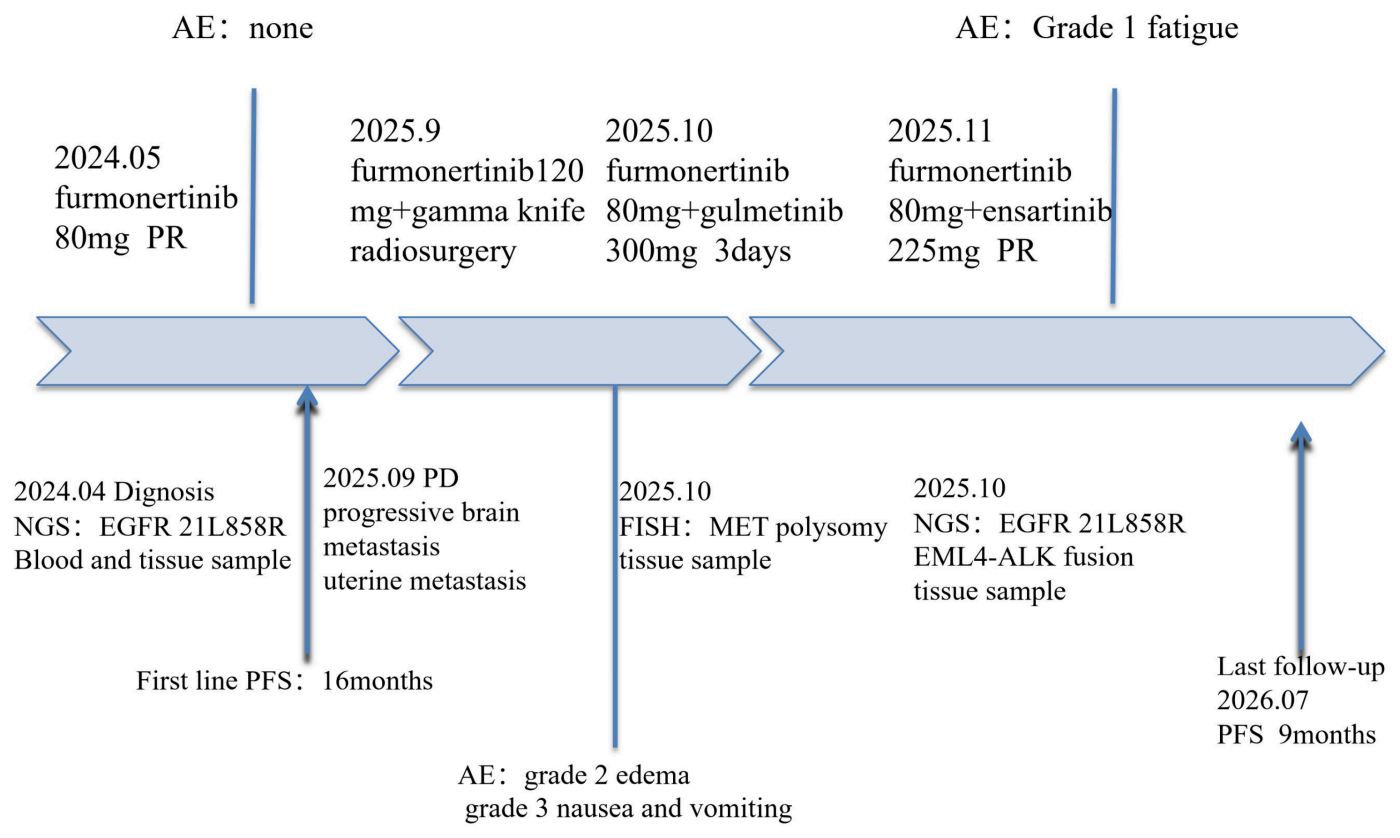
Treatment timeline of the patient in this case. PR, partial response; PD, progressive disease; PFS, progression-free survival; NA, not applicable; AE, adverse event; IHC, immunohistochemistry; NGS, next generation sequencing.

In September 2025, the patient developed lower abdominal pain. Computed tomography revealed a 6.1 × 4.9 cm heterogeneously enhancing lesion in the left pelvic cavity with obscure demarcation from the uterus, suggestive of either primary adnexal malignancy or metastatic disease. Thoracic lesions remained stable. Concomitant brain MRI indicated progression of brain metastases. The progression-free survival (PFS) under first-line furmonertinib was 16 months. The dose of furmonertinib was escalated to 120 mg once daily, and gamma knife radiosurgery was administered for brain metastases. Subsequent pelvic MRI demonstrated an enlarged uterus with diffuse myometrial thickening and abnormal signal intensity, consistent with widespread metastatic disease involving the uterus, adnexa, retroperitoneum, and bilateral iliac lymph nodes (Figure 1). Cervical core biopsy revealed poorly differentiated carcinoma, which was immunohistochemically (IHC) consistent with metastatic lung adenocarcinoma (CK7[3+], TTF-1[3+], NapsinA[2+], PAX2[1+], PAX8[−], WT1[−], p16[−], ER[−], PR[−]). PD-L1 expression was 30% (TPS), and ALK-Ventana D5F3 staining was positive. To clarify the resistance mechanism, c-MET fluorescence in situ hybridization (FISH) was performed first, which showed c-MET polysomy (mean c-MET signals per nucleus: 6.2; mean CEP7 signals per nucleus: 5.5; c-MET/CEP7 ratio: 1.13). MET polysomy represents one form of MET amplification; however, as it remains unclear whether polysomy serves as a true resistance mechanism, we initiated combination targeted therapy with glumetinib 300 mg added to furmonertinib 80 mg once daily. However, glumetinib was discontinued 3 days after treatment initiation owing to grade 2 edema and grade 3 nausea and vomiting, as defined by the CTCAE criteria. DNA-based NGS analysis of the cervical biopsy specimen identified EGFR L858R (variant allele frequency, 20.4%) and EML4-ALK fusion (variant allele frequency, 3.8%), as well as an NTRK1 exon 12 variant of uncertain significance (VUS; variant allele frequency, 3.6%). NGS did not detect MET amplification. Tumor cellularity was approximately 60%, with a mean sequencing depth of 2235× and a Q30 of 92%. Additional alterations included TP53 and IL7R mutations, MSH2 translocation, a tumor mutational burden (TMB) of 2 mutations/Mb, and microsatellite stability (MSS). Confirmatory ALK FISH verified ALK (2p23) chromosomal translocation, with positive cells accounting for 56%, establishing secondary ALK fusion as a co-existing resistance mechanism. The patient was then switched to furmonertinib 80 mg plus ensartinib 225 mg once daily. In terms of treatment-related adverse events, the patient experienced Grade 1 fatigue graded per CTCAE v5.0, with favorable overall tolerability. After one month, follow-up imaging showed marked regression: the pelvic mass decreased to 2.4 × 2.1 cm (Figure 2), the pulmonary lesion shrank from 2.2 × 1.8 cm to 1.5 × 1.3 cm, and all metastatic lymph nodes were reduced in size (Figure 3). The disease was re-evaluated as partial response (PR). The patient remained progression-free from 1 November 2025 until the last follow-up on 28 July 2026. The diagnostic, treatment and response of the patient are summarized in Figure 4.

## 3. Discussion

Lung cancer is the leading cause of cancer-related mortality worldwide, and many patients present with distant metastasis at diagnosis. The most common metastatic sites include the brain, bone, liver, and adrenal glands [8]. Notably, metastasis of lung cancer to the female reproductive system, particularly the uterus, is extremely rare. Accordingly, regular pelvic examination is not recommended in the clinical guidelines for NSCLC. Among Asian female patients with lung adenocarcinoma, the incidence of EGFR mutations can be as high as 61.1% [3,9]. EGFR mutations are correlated with more aggressive tumor progression, and relevant studies have suggested that EGFR mutations in lung adenocarcinoma may facilitate reproductive system metastasis [10]. A previous study reported 6 NSCLC cases with uterine metastasis, among whom 3 were complicated with ALK gene rearrangement and received treatment with ALK-TKIs [11]. In the present case, the patient developed metastases in female genital tract organs after 16 months of treatment with third-generation EGFR targeted agents, with newly identified ALK fusion and MET polysomy. Clinicians should be alert to the possibility of gynecological metastasis in lung cancer patients presenting with pelvic symptoms.

Triple meaningful genetic alterations in one NSCLC patient is extremely rare, with only a small number of case reports available [12,13,14,15,16]. Optimal targeted therapeutic regimens for patients with multiple concurrent gene alterations have not yet been established. Key published cases of lung cancer carrying three concomitant gene alterations are listed in Table 1, mainly involving EGFR, ALK, ROS1, MET, KRAS and HER2. Patients with advanced non-small-cell lung cancer (NSCLC) harboring EGFR mutations inevitably experience disease progression following EGFR-targeted therapy, and elucidation of the underlying resistance mechanisms is crucial for guiding subsequent anti-tumor treatment. MET polysomy, a subtype of MET amplification—an important resistance mechanism in EGFR-mutated tumors—is defined as a MET gene copy number (GCN) ≥ 5 and a MET/CEP7 ratio < 2. The MET alteration identified in this patient corresponded to chromosome 7 polysomy. The contribution of MET polysomy to drug resistance remains unclear in this case. Currently, the main detection methods for MET gene amplification include FISH and NGS. FISH is the gold standard for MET amplification testing, while NGS-based detection of MET amplification remains to be further optimized and validated. In the TATTON trial, the positive concordance rate between tissue NGS and FISH for MET amplification was 48%; further analysis revealed that the positive concordance rate for MET polysomy between NGS and FISH was only 4% [17]. This case report demonstrates that FISH testing identified MET polysomy despite negative NGS for MET amplification, underscoring the necessity of FISH as a complementary assay for MET evaluation when EGFR resistance emerges. Although the patient was administered oral MET inhibitors glumetinib, treatment was discontinued due to poor tolerability.

Oncogenic fusions, including ALK gene fusion, may also serve as a mechanism of resistance to EGFR inhibitors. Previous studies have identified gene fusions in 3–10% of cases with acquired resistance to second-line osimertinib, which may coexist with EGFR C797S mutation, BRAF mutation, and MET amplification [5]. Historically, EGFR mutations and EML4-ALK fusion were considered mutually exclusive in lung cancer patients [18,19], and co-occurrence of these two genetic alterations in the same patient has been reported mostly in isolated case studies. In one study investigating EGFR resistance mechanisms, investigators analyzed specimens from 3505 NSCLC patients harboring EGFR mutations and detected concurrent ALK fusion in 7 cases. Within this cohort, ALK fusion ranked as the second most prevalent gene fusion driving acquired resistance, only after BRAF fusion [20]. In the present case, ALK fusion was confirmed by NGS, FISH and IHC. As two serial NGS analyses were performed during the clinical course, we confirmed that the ALK fusion emerged as an acquired alteration after EGFR-TKIs treatment.

The optimal therapeutic strategy for EGFR-resistant patients with secondary ALK fusion remains unclear. Evidence suggests that patients with concurrent EGFR and ALK alterations may respond to both EGFR-TKIs and ALK-TKIs [21]. A recent literature review included 16 patients with EGFR mutations who developed acquired ALK fusions. Nine patients were treated with combined EGFR-TKI and ALK-TKI therapy, achieving an objective response rate (ORR) of 66.7%. The treatment regimen showed good tolerability, and no grade ≥ 3 adverse events were reported [7]. In the current patient, we administered ensartinib 225 mg once daily in combination with furmonertinib 80 mg, which was well-tolerated and resulted in a partial response (PR). Thus, the patient achieved clinical benefit from the addition of ALK-TKIs therapy.

This case report has several limitations. First, only NGS was performed on the primary lung lesion at baseline, and MET amplification was not further confirmed by FISH. Moreover, genetic tests were performed across multiple medical centers. Only final test results were provided, and detailed panel data were unavailable. We also had no access to information on whether fusion detection relied on DNA or RNA platforms, together with MET and ALK FISH scoring criteria and the number of counted nuclei. Second, the patient developed metastasis to the female genital system, and biopsy and genetic analysis were only performed on the uterine metastatic lesion, without re-biopsy of the primary lung tumor. Given tumor heterogeneity, genetic profiling of a single metastatic site may not fully reflect the overall genomic landscape of the patient. Third, as a retrospective case report, longer follow-up data are needed. We look forward to the conduct of prospective studies to better elucidate the clinicopathological features and optimal therapeutic strategies for EGFR-mutant NSCLC with multiple meaningful gene alterations, especially secondary ALK fusion and MET polysomy.

## 4. Conclusions

We report a rare case of gynecological metastasis in a patient with advanced EGFR exon 21 L858R mutation lung adenocarcinoma. The patient experienced disease progression after 16 months of first-line treatment with furmonertinib. NGS of the uterine metastatic lesion revealed concurrent EGFR and ALK gene alterations, while fluorescence in situ hybridization confirmed MET polysomy. We are not aware of any previous report of uterine metastasis in a female patient with advanced EGFR-mutant lung adenocarcinoma who developed acquired resistance to the third-generation EGFR-TKI furmonertinib and harbored three concomitant genetic aberrations. This finding underscores the need for pelvic examination in female lung cancer patients on targeted therapy who develop pelvic symptoms. Additionally, our case reveals that resistance mechanisms to EGFR-TKIs are highly diverse and heterogeneous. Repeat molecular profiling is essential for patients with progressive disease after EGFR-TKI administration to identify resistance drivers and guide subsequent treatment. Furthermore, additional studies are required to explore the optimal combination targeted regimens for patients carrying multiple concurrent oncogenic alterations.

## Figures and Tables

**Table 1:** Characteristics of patients with 3 driver gene mutations in NSCLC.

Authors (Year)	Study Type	Age/Sex	Co-Mutation Gene1	Co-Mutation Gene2	Co-Mutation Gene3	First-Line Targeted Therapy (PFS)	Subsequent-Line Targeted Therapy (PFS)
Rossing H.H. et al. (2013) [12]	Case	61/M	EGFR L862R	EML4-ALK	KRAS G13D	crizotinib (8 months)	N/A
Ju L. et al. (2016) [13]	Case	53/M	EGFR 19del	ROS1 fusion	KRAS G12A	crizotinib (1 months)	Icotinib (8 months)
Zhuang X. et al. (2019) [14]	Retrospective study 2 cases	52/M	EGFR	ROS1	KRAS	Gefitinib 12.7 months	N/A
		53/M	EGFR	ROS1	KRAS	crizotinib	Icotinib Total 27.5 months
Yin Y. et al. (2021) [15]	Case	59/M	EGFR 19del	EML4-ALK	MET amplification	Gefitinib 19 months	Osimertinib (6 months) → crizotinib+Gefitinib (12 months)
Li W. et al. (2023) [16]	Retrospective study 3 cases	N/A	EGFR	Her2	MET	N/A	N/A
			ALK	Her2	MET		
			EGFR	ALK	ROS1		

## Data Availability

Data are contained within the article.
